# Prize Essays

**Published:** 1851-10

**Authors:** 


### PRIZE ESSAYS.

We take pleasure in republishing the following circular:—

"*American Medical Asssociation. Prize Essays.*—At a meeting of the American Medical Association, held in Charleston, S. C., in May last, the undersigned were appointed a committee to receive and examine such voluntary communications on subjects connected with medical science as individuals might see fit to make, and to award a prize to any number of them not exceeding five, if they should be regarded as entitled to such a distinction.

"To carry into effect the intentions of the association, notice is hereby given that all such communications must be sent, post-paid, on or before the first day of April, 1852, to Geo. Hayward, M. D., Boston, Mass. Each communication must be accompanied by a packet containing the name of the author, which will not be opened unless the accompanying communication be deemed worthy of a prize. The authors of the unsuccessful papers may receive them on application to the committee, at any time after the first of June, 1852; and the successful ones, it is understood, will be printed in the Transactions of the Association."

George Hayward, *Boston.*
J. B. S. Jackson, "
D. H. Storer, "
Jacob Bigelow, "
Usher Parsons, *Providence, R. I.*

Boston, Aug. 20, 1851.

